# Synthesis, crystal structure and reactivity of bis­(μ-2-methyl­pyridine *N*-oxide-κ^2^ *O*:*O*)bis­[di­bromido­(2-methyl­pyridine *N*-oxide-κ*O*)cobalt(II)] butanol monosolvate

**DOI:** 10.1107/S2056989023008228

**Published:** 2023-10-03

**Authors:** Christian Näther, Inke Jess

**Affiliations:** a Institut für Anorganische Chemie, Universität Kiel, Germany; University of Kentucky, USA

**Keywords:** crystal structure, synthesis, thermoanalytical investigations, cobalt thio­cyanate, 2-methyl­pyridine *N*-oxide

## Abstract

Reaction of CoBr_2_ with 2-methyl­pyridine *N*-oxide in *n*-butanol leads to the formation of the title compound, [CoBr_2_]_2_(2-methyl­pyridine *N*-oxide)_4_·*n*-butanol or [Co_2_Br_4_(C_6_H_7_NO)_4_]·C_4_H_10_O. The asymmetric unit of the title compound consists of one Co^II^ cation as well as two bromide anions and two 2-methyl­pyridine *N*-oxide coligands in general positions and one *n*-butanol mol­ecule that is disordered around a center of inversion. The Co^II^ cations are fivefold coordinated by two bromide anions and one terminal as well as two bridging 2-methyl­pyridine *N*-oxide and linked by two symmetry-related μ-1,1(*O*,*O*) 2-methyl­pyridine *N*-oxide coligands into dinuclear units that are located on centers of inversion. In the crystal structure, the dinuclear units are also connected *via* pairs of C—H⋯Br hydrogen bonds into chains that elongate in the *b*-axis direction. The *n*-butanol mol­ecules are located between the chains and are linked *via* O—H⋯Br hydrogen bonds each to one chain. Powder X-ray diffraction (PXRD) measurements reveal that a pure phase has been obtained. Measurements using thermogravimetry and differential thermoanalysis shows one mass loss up to 523 K, in which the *n*-butanol mol­ecules are removed. PXRD measurements of the residue obtained after *n*-butanol removal shows that a completely different crystalline phase has been obtained and IR investigations indicate significant structural changes in the Co coordination.

## Abstract 2

The crystal structure of the title compound consists of dinuclear complexes, in which the Co^II^ cations are fivefold coordinated and linked by centrosymmetric pairs of μ-1,1(*O*,*O*)-bridging 2-methyl­pyridine *N*-oxide coligands.

## 1. Chemical context

Transition-metal halide coordination compounds show a large structural variability because the halide anions can act as terminal or bridging ligands (Peng *et al.*, 2010 ▸). This can lead to the formation of metal–halide substructures of different dimensionality, like, e.g. mono- and dinuclear units, chains, double chains or layered compounds, that can be further connected by the use of bridging coligands (Peng *et al.*, 2010 ▸ and Näther *et al.*, 2007 ▸). In general the dimensionality of the network predominantly depends on the ratio between the transition metal halide and the coligand. Compounds with a large ratio usually show a low dimensionality and form discrete units, whereas the dimensionality of the metal halide substructure increases with decreasing amount of the coligands (Näther *et al.*, 2001 ▸; Näther and Jess, 2001 ▸). Even if in the majority of cases such compounds were prepared in solution, we have found that upon heating, the coligand-rich compounds lose their ligands stepwise, which leads to the formation of compounds with higher dimensionality (Näther *et al.*, 2001 ▸; Näther & Jess, 2004 ▸). In the beginning, this approach was used for the preparation of Cu^I^ compounds (Näther *et al.*, 2001 ▸, 2002 ▸), but later it was expanded to compounds with twofold positively charged cations, because even such compounds show a variety of structures of different dimensionality (Näther *et al.*, 2007 ▸). In this context, it is noted that this thermal ligand removal can also be used for the synthesis of compounds with different anions such as, for example, thio- or seleno­cyanates (Werner *et al.*, 2015 ▸; Rams *et al.*, 2020 ▸). 
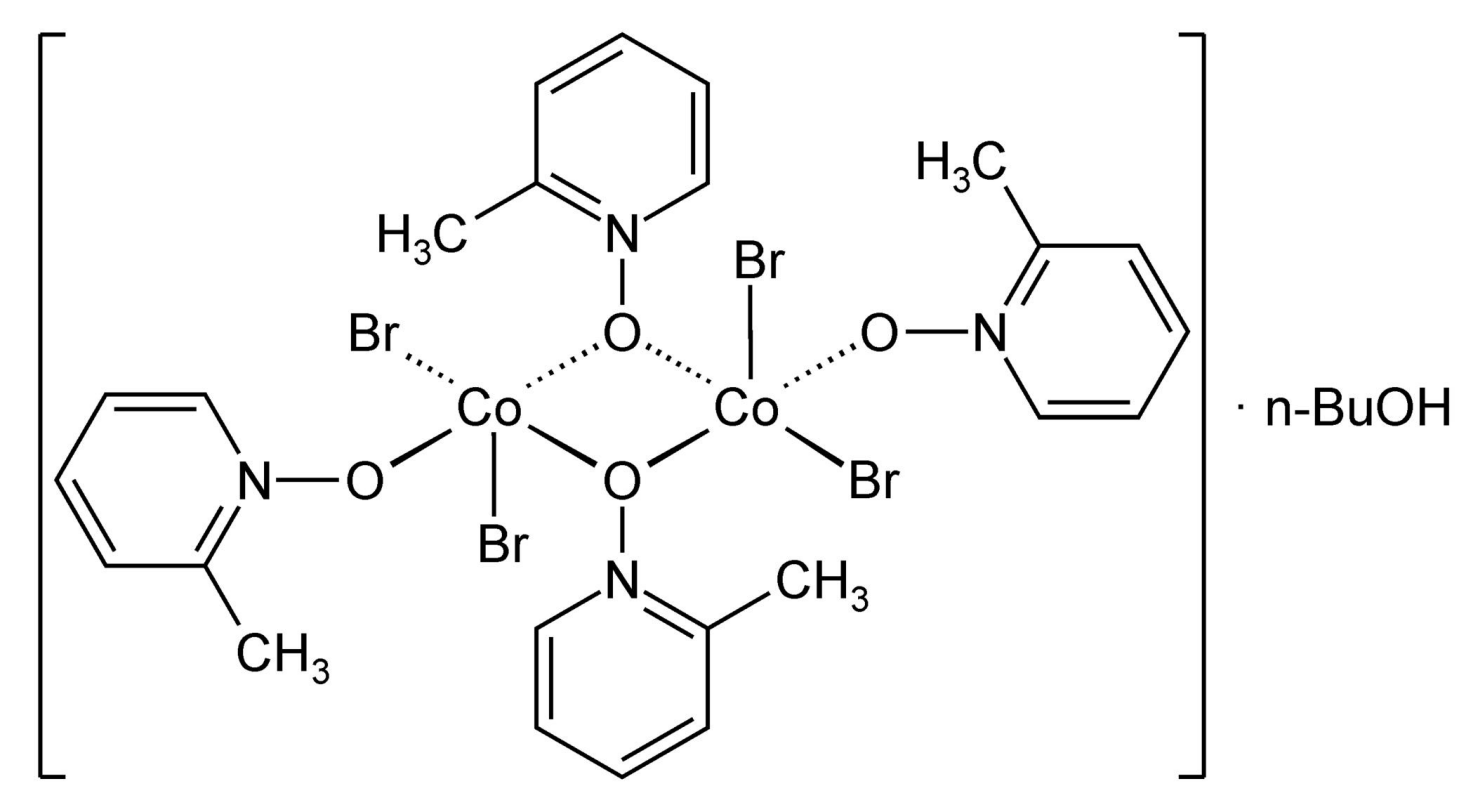


In recent work, we exclusively used N-donor coligands that in most cases consist of pyridine derivatives. Therefore, the question arose whether this method could also be expanded to other coligands and in this context we became inter­ested in pyridine *N*-oxide derivatives, because in contrast to pyridine derivatives they can act as terminal but also as bridging ligands. In this context, it would also be of inter­est if they show a similar thermal reactivity to that of the pyridine analogs. It is also noted that some transition-metal halide compounds with pyridine *N*-oxide derivatives have already been reported in the literature. In the course of our systematic work we used 2-methyl­pyridine *N*-oxide as ligand, for which some transition-metal halide compounds have already been reported in the literature. Compounds based on cobalt(II) are not reported, which also might be of inter­est in terms of magnetic properties. In the first experiments we reacted CoBr_2_ with 2-meth­yl­pyridine in different solvents and from *n*-butanol we obtained blue-colored crystals that were identified by single-crystal structure analysis.

## 2. Structural commentary

**Figure 1:**
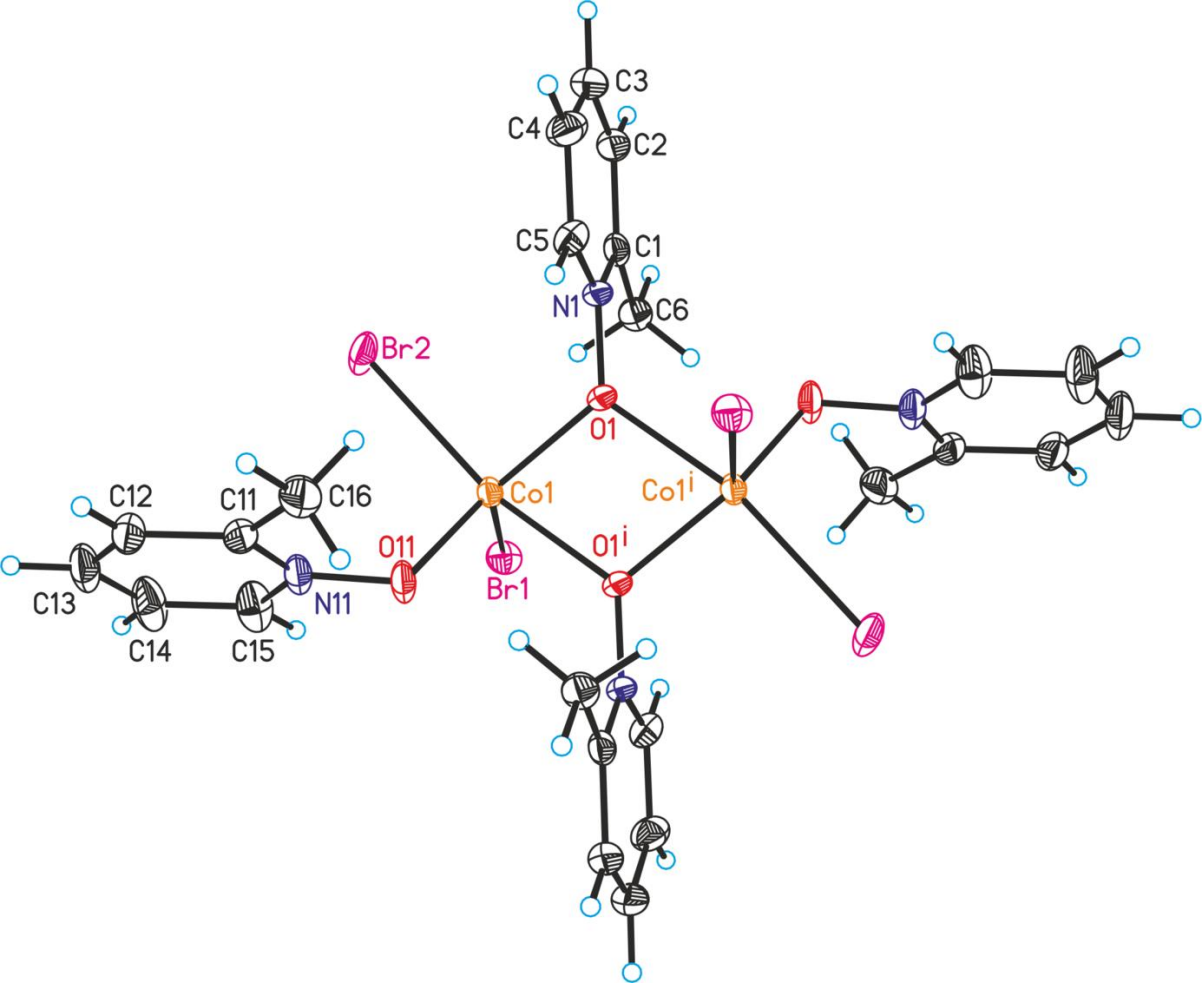
Crystal structure of the dinuclear unit in the title compound with labeling and displacement ellipsoids drawn at the 50% probability level. Symmetry code: (i) −*x* + 1, −*y* + 1, −*z* + 1.

**Figure 2:**
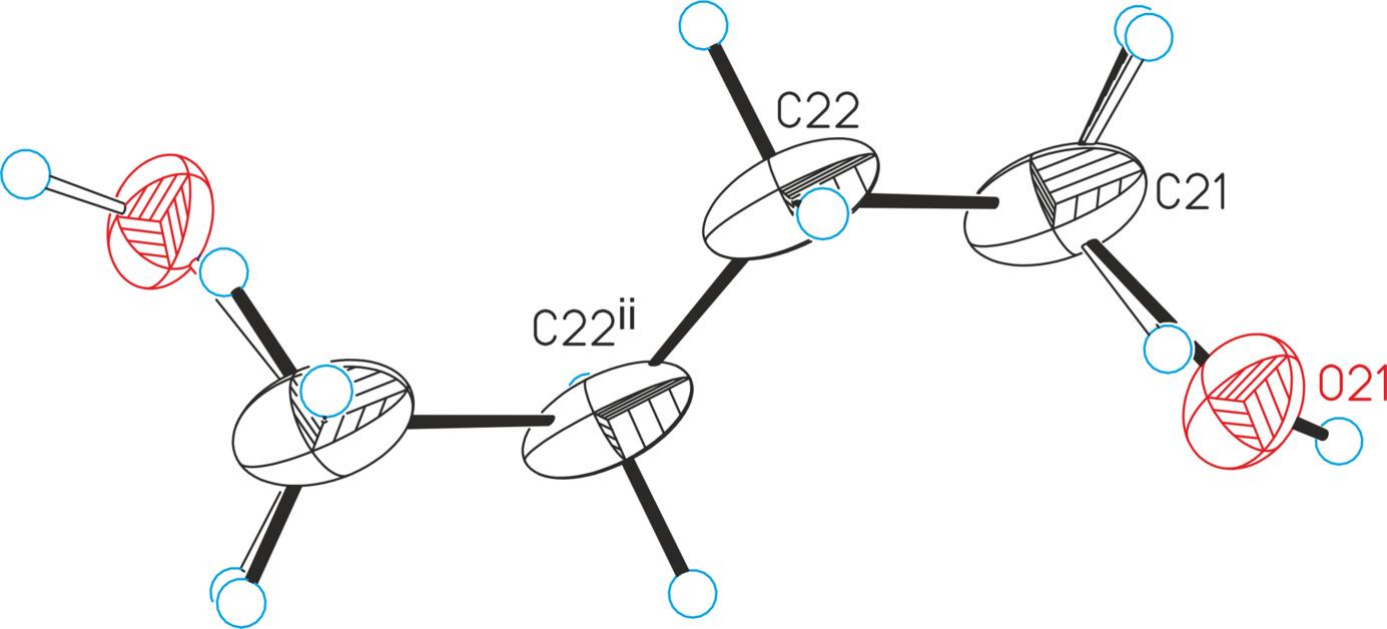
Crystal structure of the disordered butanol mol­ecule in the title compound with labeling and displacement ellipsoids drawn at the 50% probability level. Symmetry code: (ii) −*x* + 1, −*y*, −*z*. The disorder is shown with full and open bonds.

The asymmetric unit of the title compound, [CoBr_2_]_2_(2-meth­yl­pyridine *N*-oxide)_4_·*n*-butanol, consists of one Co^II^ cation as well as two bromide anions and two 2-methyl­pyridine *N*-oxide coligands in general positions (Fig. 1 ▸) and one *n*-butanol mol­ecule that is located on a center of inversion and is therefore disordered due to symmetry (Fig. 2 ▸). This disorder remains constant if the refinement is performed in the space group *P*1 (see *Refinement*). The Co^II^ cations are fivefold coordinated by two bromide anions as well as one terminal and two bridging 2-methyl­pyridine *N*-oxide coligands. From the bond lengths and angles it is obvious that an irregular Co coordination is present, that is in between that of a trigonal bipyramid and a tetra­gonal pyramid (Table 1 ▸). Each of the two Co^II^ cations is linked by two μ-1,1(*O*,*O*) 2-methyl­pyridine *N*-oxide coligands into dinuclear units that are located on centers of inversion (Fig. 1 ▸). The distance between the two Co^II^ cations within the four-membered Co_2_O_2_ rings amounts to 3.4196 (7) Å and the rings are planar.

In this context, it is noted that a compound with the composition [CuCl_2_]_2_(4-methyl­pyridine *N*-oxide)_4_ is reported, which shows a structure that is analogous to that of the title compound (refcode CMPYUC; Johnson & Watson, 1971*a* ▸). A similar structure is also observed for [MnBr_2_]_2_(4-methyl­pyridine *N*-oxide)_4_(MeOH)_2_ that consists of the same dimeric units but each of the Mn^II^ cations is additionally coordinated by a methanol mol­ecule, leading to an octa­hedral coordination (refcode VONHOY; Lynch *et al.*, 2019 ▸). Such a structure is also reported with 3-methyl­pyridine *N*-oxide (see *Database survey*). Finally, a related dinuclear complex with a tetra­hedral coordination is observed in [CuCl_2_]_2_(4-methyl­pyridine *N*-oxide)_2_ (refcode QQQBWJ; Kidd *et al.*, 1967 ▸) and [CuBr_2_]_2_(4-methyl­pyridine *N*-oxide)_2_ (refcode DURYIY; Nepveu *et al.*, 1986 ▸), where the two terminal pyridine *N*-oxide ligands are missing.

## 3. Supra­molecular features

**Figure 3:**
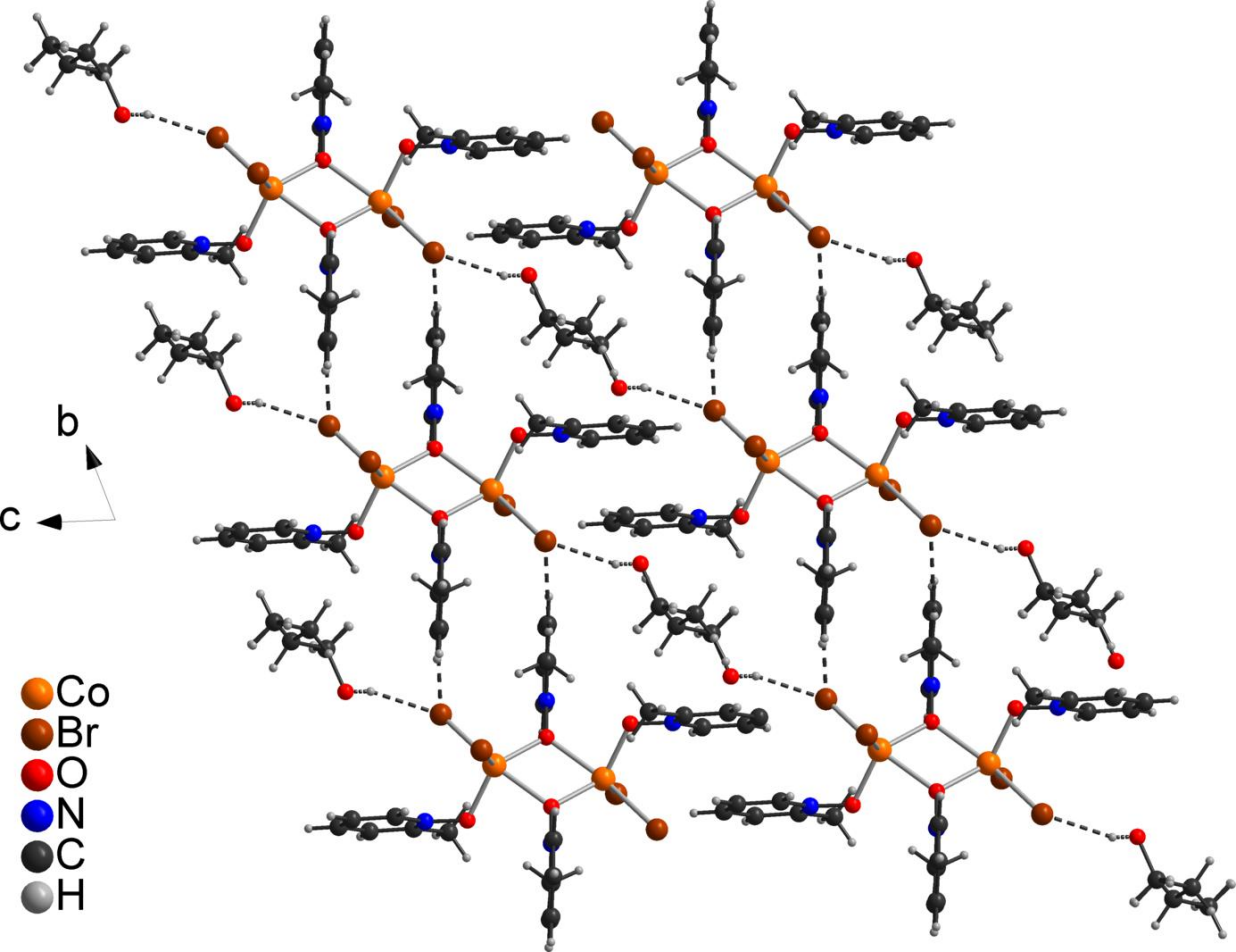
Crystal structure of compound **1** viewed along the crystallographic *a*-axis. Inter­molecular C—H⋯Br and C—H⋯O hydrogen bonding is shown as dashed lines. Please note that the *n*-butanol mol­ecule is disordered around centers of inversion. For the *n*-butanol mol­ecules between the chains the disorder is not removed, whereas for the *n*-butanol mol­ecules left and right from the chains each one O atom is arbitrarily removed.

In the crystal structure of compound **1**, a number of inter­molecular C—H⋯O and C—H⋯Br contacts are observed but most of them show angles far from linearity, indicating that these correspond to very weak inter­actions (Table 2 ▸). However, a few of them show distances and angles that point to inter­molecular hydrogen bonding and if they are considered as significant inter­actions, the discrete complexes are connected into chains that propagate along the crystallographic *a*-axis direction (Fig. 3 ▸ and Table 2 ▸). The *n*-butanol mol­ecules are located between these chains and are linked *via* O—H⋯Br hydrogen bonding to the chains. Because they are disordered around a center of inversion, in the middle of Fig. 3 ▸ it appears that they inter­connect to neighboring chains, but in fact they are always arbitrarily connected to only one of these chains (Fig. 3 ▸).

## 4. Thermoanalytical and powder X-ray powder investigations

**Figure 4:**
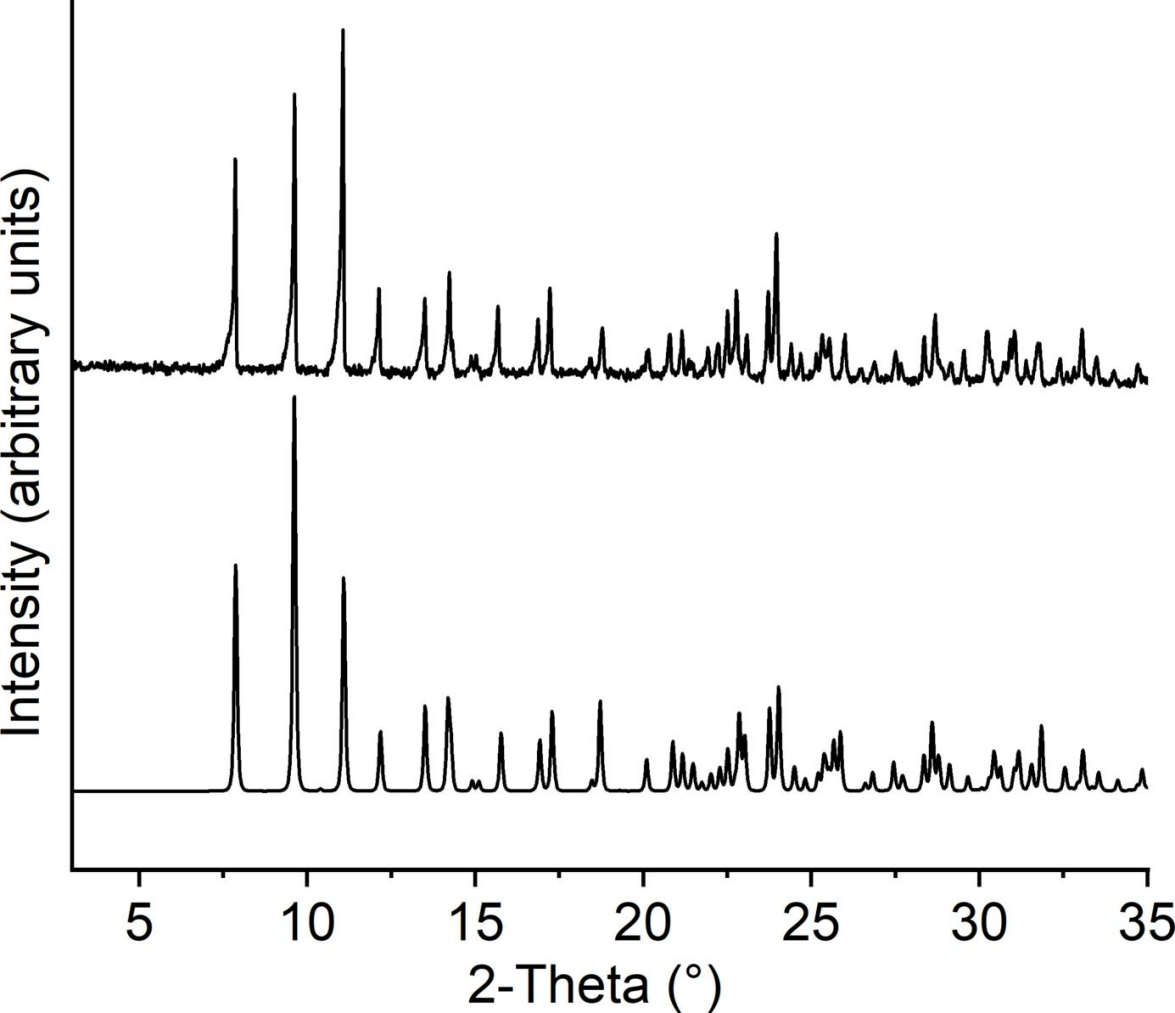
Experimental (top) and calculated powder pattern (bottom) for the title compound.

Comparison of the experimental powder pattern of the title compound with that calculated from single-crystal data using structural data obtained at room temperature proves that a pure crystalline phase has been obtained (Fig. 4 ▸).

To investigate the thermal properties of the title compound including solvent removal, measurements using simultaneous differential thermoanalysis and thermogravimetry (DTA-TG) were performed. Upon heating, two mass losses are observed that are accompanied by endothermic events in the DTA curve (Fig. S1). From the DTG curve, it is obvious that the first mass loss is well resolved, which is not the case for the second mass loss. Moreover, the sample mass decreases continuously upon further heating, with no distinct step that points to the formation of a further compound (Fig. S1). The experimental mass loss of 8.9% in the first mass loss is in rough agreement with that calculated for the removal of the butanol mol­ecules (Δ*m*_calc_ = −7.8%), indicating the formation of a new compound with the composition CoBr_2_(2-methyl­pyridine *N*-oxide)_2_. It is noted that after the formation of the new inter­mediate compound there is an endothermic event where the sample mass does not change, indicating that the overall reaction is more complex.

PXRD investigations of the residue obtained after the first mass loss prove that a highly crystalline and completely different phase has been obtained (please compare Fig. 1 ▸ and S2) and IR investigations reveal significant differences, indicating that the Co coordination has changed (Figs. S3 and S4).

Finally, from the TG curve it is obvious that the first mass loss starts at very low temperature, indicating that the compound had already decomposed at room temperature (Fig. S1). Therefore, a freshly prepared batch of the title compound was stored for 60 h at room temperature and afterwards was investigated by PXRD, which proved that a transformation into the new crystalline phase obtained by solvent removal at elevated temperatures is obtained (Fig. S5).

## 5. Database survey

No crystal structures of cobalt halide compounds with methyl­pyridine *N*-oxide are reported in the CSD (version 5.43, last update March 2023; Groom *et al.*, 2016 ▸) but some compounds with other transition-metal cations are known.

These include CuCl_2_(2-methyl­pyridine *N*-oxide)_2_ and ZnCl_2_(2-methyl­pyridine *N*-oxide)_2_, which form discrete complexes in which the metal cations are tetra­hedrally coordinated (refcodes QQQBVY and QQQBXY; Kidd *et al.*, 1967 ▸) as well as [CuCl_2_]_3_(2-methyl­pyridine *N*-oxide)_2_(H_2_O)_2_ (refcode PIOCUA; Sager & Watson, 1968 ▸).

One compound with the composition MnCl_2_(2-methyl­pyridine *N*-oxide)(H_2_O) is also reported (refcode VEJMAB; Kang *et al.*, 2017 ▸). In this compound, the Mn^II^ cations are octa­hedrally coordinated by one terminal chloride anion, one terminal water mol­ecule as well as two bridging chloride anions and two bridging 2-methyl­pyridine *N*-oxide coligands. The cations are linked by pairs of alternating μ-1,1(*O*,*O*)-bridging 2-methyl­pyridine *N*-oxide coligands and each of the two μ(1,1) chloride anions into linear chains.

In [MnBr_2_]_2_(2-methyl­pyridine *N*-oxide)_2_(H_2_O)_4_ bis­(2-methyl­pyridine *N*-oxide) solvate, each Mn^II^ cation is octa­hedrally coordinated by two water mol­ecules, two bromine atoms and two bridging 2-methyl­pyridine *N*-oxide coligands (refcode VONHEO; Lynch *et al.*, 2019 ▸). Each of the two Mn^II^ cations is linked by two μ-1,1(*O*,*O*)-bridging 2-methyl­pyridine *N*-oxide ligands into dinuclear complexes.

There are additional compounds with, for example, protonated 2-methyl­pyridine *N*-oxide cations and tetra­chloro aurate (refcode CICBIZ; Hussain & Aziz al-Hamound, 1984 ▸) as well as Co(ClO_4_)_2_(2-methyl­pyridine *N*-oxide)_5_ (refcodes PICOCO and PICOCO01; Coyle & Ibers, 1970 ▸ and Bertini *et al.*, 1975 ▸).

With 3-methyl­pyridine *N*-oxide and 4-methyl­pyridine *N*-oxide, no cobalt halide compounds are known but one compound with an essentially identical structure is reported with CuCl_2_ and 4-methyl­pyridine *N*-oxide; this is mentioned in the *Structural commentary* (refcode CMPYUC; Johnson & Watson, 1971*a* ▸).

With 4-methyl­pyridine *N*-oxide, discrete tetra­hedral complexes with the composition *M*Cl_2_(4-methyl­pyridine *N*-oxide)_2_ with *M* = Cu and Zn (refcodes CMPOCU, CMPOCU01 and QQQBXG; Johnson & Watson, 1971*b* ▸ and Kidd *et al.*, 1967 ▸) and ZnI_2_(4-methyl­pyridine *N*-oxide) are reported (refcode SANRUV; Shi *et al.*, 2005 ▸).

Discrete tetra­hedral complexes with CuCl_2_ and ZnCl_2_ are also reported with 3-methyl­pyridine *N*-oxide (refcodes QQQBWA, QQQBWA01 and QQQBXM; Kidd *et al.*, 1967 ▸). Dinuclear complexes with 3-methyl­pyridine *N*-oxide and fourfold or sixfold metal coordination are observed in [CuCl_2_]_2_(3-methyl­pyridine *N*-oxide)_2_ (refcode QQQBWG; Kidd *et al.*, 1967 ▸) and in MnCl_2_]_2_(3-methyl­pyridine *N*-oxide)_2_(H_2_O)_2_ (refcode VEJMEF; Kang *et al.*, 2017 ▸). A dinuclear complex similar to that of the title compound but with an octa­hedral coordination is reported with MnBr_2_ and 3-methyl­pyridine *N*-oxide (refcode VONHIS; Lynch *et al.*, 2019 ▸). Finally, there are some additional compounds with other metal cations that are similar to that of the title compound (see *Structural commentary*).

## 6. Synthesis and crystallization

CoBr_2_ (97%) was purchased from Alfa Aesar, 2-methyl­pyridine *N*-oxide (98%) and anhydrous *n*-butanol was purchased from Thermo Scientific.

**Synthesis:**

0.5 mmol (109 mg) of CoBr_2_ and 2 mmol (218.0 mg) of 2-methyl­pyridine in 1 mL of anhydrous *n*-butanol were heated for 2 d at 388 K. After cooling to room temperature, blue-colored block-like crystals were obtained.

An IR spectrum of the title compound can be found in Fig. S4.

Finally, it is noted that because of the disorder of the *n*-butanol mol­ecule we also tried to prepare a compound with 1,4-butane­diol instead of butanol, which should occupy the same position as that of the *n*-butanol mol­ecule, but microcrystalline powders were always obtained that showed a powder pattern identical to that of the residues obtained by solvent removal from the title compound.

**Experimental details:**

The data collection for single-crystal structure analysis was performed using an XtaLAB Synergy, Dualflex, HyPix diffractometer from Rigaku with Cu *Kα* radiation.

Thermogravimetry and differential thermoanalysis (TG-DTA) measurements were performed in a dynamic nitro­gen atmosphere in Al_2_O_3_ crucibles using a STA-PT 1000 thermobalance from Linseis. The instrument was calibrated using standard reference materials.

The PXRD measurements were performed with a Stoe Transmission Powder Diffraction System (STADI P) equipped with a MYTHEN 1K detector and a Johansson-type Ge(111) monochromator using Cu *Kα_1_* radiation (λ = 1.540598 Å).

The IR spectra were measured using an ATI Mattson Genesis Series FTIR Spectrometer, control software: *WINFIRST*, from ATI Mattson.

## 7. Refinement

Crystal data, data collection and structure refinement details are summarized in Table 3 ▸. The C—H hydrogen atoms were positioned with idealized geometry (methyl H atoms allowed to rotate but not to tip) and were refined isotropically with *U*_iso_(H) = 1.2*U*_eq_(C) (1.5 for methyl hydrogen atoms) using a riding model.

As already mentioned, the *n*-butanol mol­ecule is disordered around a center of inversion, which is located exactly in the middle of the central C—C bond. Therefore, the generation of the symmetry-equivalent terminal atom formally lead to a mol­ecule with a six-membered chain. However, the assignment of oxygen to the terminal atom lead to a much too high anisotropic displacement parameter, which decreased to a reasonable value if the site occupation is reduced to 0.5. After anisotropic refinement, only one electron-density peak is observed close to the O atom, which can clearly be assigned to the missing O—H hydrogen atom. For the C—O bond lengths, a restraint was used because otherwise a too long bond length was obtained. This presumably can be traced back to some disordering, because of the superposition of *n*-butanol mol­ecules that are connect to different chains, which is also reflected in slightly enhanced components of the anisotropic displacement parameters of the C atoms of these mol­ecules.

Finally it is noted that the disorder remains constant if the refinement is performed in the space group *P*1 and that no super structure reflections are visible that might point to a larger unit cell.

## Supplementary Material

Crystal structure: contains datablock(s) I. DOI: 10.1107/S2056989023008228/pk2697sup1.cif

Structure factors: contains datablock(s) I. DOI: 10.1107/S2056989023008228/pk2697Isup2.hkl

DTG, TG and DTA curve of the title compound measured with 4C/min in a nitrogen atmosphere. DOI: 10.1107/S2056989023008228/pk2697sup3.png

Experimental powder pattern of the residue obtained after the first mass loss in a DTA-TG measurement of the title compound. DOI: 10.1107/S2056989023008228/pk2697sup4.png

IR spectrum of the residue obtained after the first mass loss in a DTA-TG measurement of the title compound. The wavenumbers of the most prominent vibrations are given. DOI: 10.1107/S2056989023008228/pk2697sup5.png

IR spectrum of the title compound. The wavenumbers of the most prominent vibrations are given. DOI: 10.1107/S2056989023008228/pk2697sup6.png

Experimental powder pattern of the residue obtained after storage of the title compound for 60 h at room-temperature. DOI: 10.1107/S2056989023008228/pk2697sup7.png

CCDC reference: 2295983

Additional supporting information:  crystallographic information; 3D view; checkCIF report

## Figures and Tables

**Table 1:** Selected geometric parameters (Å, °)

Co1—Br1	2.4312 (4)	Co1—O1^i^	2.1968 (15)
Co1—Br2	2.5217 (4)	Co1—O11	1.9732 (17)
Co1—O1	1.9964 (16)		
			
Br1—Co1—Br2	101.355 (16)	O11—Co1—Br2	97.54 (5)
O1—Co1—Br1	116.56 (5)	O11—Co1—O1^i^	83.12 (6)
O1^i^—Co1—Br1	95.39 (4)	O11—Co1—O1	126.08 (7)
O1—Co1—Br2	94.52 (4)	Co1—O1—Co1^i^	109.18 (7)
O1^i^—Co1—Br2	161.54 (4)	N1—O1—Co1	125.78 (12)
O1—Co1—O1^i^	70.82 (7)	N1—O1—Co1^i^	124.80 (12)
O11—Co1—Br1	112.08 (6)		

Symmetry code: (i) 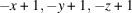 .

**Table 2:** Hydrogen-bond geometry (Å, °)

*D*—H⋯*A*	*D*—H	H⋯*A*	*D*⋯*A*	*D*—H⋯*A*
C5—H5⋯Br1^ii^	0.95	3.12	3.704 (2)	121
C5—H5⋯Br1^i^	0.95	2.98	3.531 (2)	118
C6—H6*A*⋯O11^i^	0.98	2.45	3.259 (3)	140
C6—H6*B*⋯Br2^iii^	0.98	2.88	3.852 (2)	172
C6—H6*C*⋯Br1	0.98	3.02	3.845 (2)	142
C12—H12⋯Br2^iv^	0.95	2.99	3.676 (3)	130
C12—H12⋯O21^iv^	0.95	2.55	3.441 (6)	156
C14—H14⋯Br1^v^	0.95	3.11	3.747 (3)	126
C16—H16*C*⋯Br1^ii^	0.98	3.13	3.725 (3)	121
O21—H21⋯Br2	0.84	2.43	3.244 (4)	162

Symmetry codes: (i) 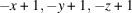 ; (ii) 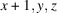 ; (iii) 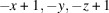 ; (iv) 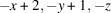 ; (v) 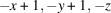 .

**Table 3:** Experimental details

Crystal data	
Chemical formula	[Co_2_Br_4_(C_6_H_7_NO)_4_]·C_4_H_10_O
*M*_r_	948.12
Crystal system, space group	Triclinic, *P* 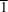
Temperature (K)	100
*a*, *b*, *c* (Å)	8.0900 (1), 9.5772 (1), 12.2400 (1)
α, β, γ (°)	70.242 (1), 76.004 (1), 83.860 (1)
*V* (Å^3^)	865.69 (2)
*Z*	1
Radiation type	Cu *K*α
μ (mm^−1^)	13.26
Crystal size (mm)	0.15 × 0.03 × 0.03

Data collection	
Diffractometer	XtaLAB Synergy, Dualflex, HyPix
Absorption correction	Multi-scan (*CrysAlis PRO*; Rigaku OD, 2022 ▸)
*T*_min_, *T*_max_	0.721, 1.000
No. of measured, independent and observed [*I* > 2σ(*I*)] reflections	18480, 3705, 3686
*R*_int_	0.021
(sin θ/λ)_max_ (Å^−1^)	0.639

Refinement	
*R*[*F* ^2^ > 2σ(*F* ^2^)], *wR*(*F* ^2^), *S*	0.024, 0.065, 1.09
No. of reflections	3705
No. of parameters	204
No. of restraints	1
H-atom treatment	H-atom parameters constrained
Δρ_max_, Δρ_min_ (e Å^−3^)	0.50, −0.59

Computer programs: *CrysAlis PRO* (Rigaku OD, 2022 ▸), *SHELXT2014/4* (Sheldrick, 2015*a* ▸), *SHELXL2016/6* (Sheldrick, 2015*b* ▸), *DIAMOND* (Brandenburg & Putz, 1999 ▸) and *publCIF* (Westrip, 2010 ▸).

## supplementary crystallographic information

### Crystal data

**Table:**

[Co_2_Br_4_(C_6_H_7_NO)_4_]·C_4_H_10_O	*Z* = 1
*M**_r_* = 948.12	*F*(000) = 468
Triclinic, *P*1	*D*_x_ = 1.819 Mg m^−^^3^
*a* = 8.0900 (1) Å	Cu *K*α radiation, λ = 1.54184 Å
*b* = 9.5772 (1) Å	Cell parameters from 14929 reflections
*c* = 12.2400 (1) Å	θ = 3.9–79.9°
α = 70.242 (1)°	µ = 13.26 mm^−^^1^
β = 76.004 (1)°	*T* = 100 K
γ = 83.860 (1)°	Block, dark blue
*V* = 865.69 (2) Å^3^	0.15 × 0.03 × 0.03 mm

### Data collection

**Table:**

XtaLAB Synergy, Dualflex, HyPix diffractometer	3705 independent reflections
Mirror monochromator	3686 reflections with *I* > 2σ(*I*)
Detector resolution: 10.0000 pixels mm^-1^	*R*_int_ = 0.021
ω scans	θ_max_ = 80.2°, θ_min_ = 3.9°
Absorption correction: multi-scan (CrysAlisPro; Rigaku OD, 2022)	*h* = −10→10
*T*_min_ = 0.721, *T*_max_ = 1.000	*k* = −12→12
18480 measured reflections	*l* = −12→15

### Refinement

**Table:**

Refinement on *F*^2^	Hydrogen site location: inferred from neighbouring sites
Least-squares matrix: full	H-atom parameters constrained
*R*[*F*^2^ > 2σ(*F*^2^)] = 0.024	*w* = 1/[σ^2^(*F*_o_^2^) + (0.0306*P*)^2^ + 1.2686*P*] where *P* = (*F*_o_^2^ + 2*F*_c_^2^)/3
*wR*(*F*^2^) = 0.065	(Δ/σ)_max_ < 0.001
*S* = 1.09	Δρ_max_ = 0.50 e Å^−^^3^
3705 reflections	Δρ_min_ = −0.59 e Å^−^^3^
204 parameters	Extinction correction: *SHELXL2016/6* (Sheldrick, 2015b), Fc^*^=kFc[1+0.001xFc^2^λ^3^/sin(2θ)]^-1/4^
1 restraint	Extinction coefficient: 0.00106 (14)
Primary atom site location: dual	

### Special details

**Table:**

Geometry. All esds (except the esd in the dihedral angle between two l.s. planes) are estimated using the full covariance matrix. The cell esds are taken into account individually in the estimation of esds in distances, angles and torsion angles; correlations between esds in cell parameters are only used when they are defined by crystal symmetry. An approximate (isotropic) treatment of cell esds is used for estimating esds involving l.s. planes.

### Fractional atomic coordinates and isotropic or equivalent isotropic displacement parameters (Å^2^)

**Table:**

	*x*	*y*	*z*	*U*_iso_*/*U*_eq_	Occ. (<1)
Co1	0.52210 (5)	0.46947 (4)	0.36734 (3)	0.01558 (10)	
Br1	0.24435 (3)	0.41656 (3)	0.34944 (2)	0.02233 (8)	
Br2	0.71203 (4)	0.28893 (3)	0.28069 (2)	0.02820 (9)	
O1	0.5761 (2)	0.38106 (17)	0.52800 (14)	0.0163 (3)	
N1	0.6713 (2)	0.2555 (2)	0.56405 (17)	0.0161 (4)	
C1	0.5945 (3)	0.1227 (3)	0.60505 (19)	0.0183 (4)	
C2	0.6944 (3)	−0.0046 (3)	0.6432 (2)	0.0238 (5)	
H2	0.644348	−0.099275	0.673735	0.029*	
C3	0.8660 (4)	0.0060 (3)	0.6368 (2)	0.0279 (5)	
H3	0.933382	−0.081032	0.663242	0.033*	
C4	0.9388 (3)	0.1438 (3)	0.5918 (2)	0.0264 (5)	
H4	1.056708	0.152377	0.586252	0.032*	
C5	0.8382 (3)	0.2689 (3)	0.5550 (2)	0.0212 (5)	
H5	0.886759	0.364257	0.523258	0.025*	
C6	0.4125 (3)	0.1211 (3)	0.6032 (2)	0.0211 (5)	
H6A	0.345495	0.183462	0.647998	0.032*	
H6B	0.372568	0.019130	0.639542	0.032*	
H6C	0.398996	0.159651	0.520671	0.032*	
O11	0.6147 (2)	0.65388 (19)	0.24397 (14)	0.0243 (4)	
N11	0.6946 (3)	0.6523 (2)	0.13423 (17)	0.0234 (4)	
C11	0.8634 (3)	0.6742 (3)	0.0986 (2)	0.0254 (5)	
C12	0.9411 (4)	0.6781 (3)	−0.0169 (2)	0.0318 (6)	
H12	1.059812	0.694910	−0.045090	0.038*	
C13	0.8485 (4)	0.6581 (3)	−0.0914 (2)	0.0346 (7)	
H13	0.903094	0.660251	−0.169909	0.041*	
C14	0.6774 (4)	0.6351 (3)	−0.0508 (2)	0.0369 (7)	
H14	0.611936	0.621342	−0.101029	0.044*	
C15	0.6004 (4)	0.6320 (3)	0.0638 (2)	0.0320 (6)	
H15	0.481643	0.615737	0.092873	0.038*	
C16	0.9546 (4)	0.6917 (4)	0.1847 (3)	0.0347 (6)	
H16A	0.911410	0.781415	0.204957	0.052*	
H16B	1.076871	0.700157	0.148968	0.052*	
H16C	0.935641	0.604983	0.256956	0.052*	
O21	0.6568 (6)	0.1984 (4)	0.0597 (4)	0.0427 (10)	0.5
H21	0.673583	0.200477	0.124200	0.064*	0.5
C21	0.6912 (6)	0.0492 (4)	0.0530 (3)	0.0562 (10)	
H21A	0.658475	−0.023433	0.133764	0.067*	0.5
H21B	0.814941	0.035705	0.023128	0.067*	0.5
H21C	0.665943	−0.027068	0.131402	0.067*	0.5
H21D	0.656102	0.147024	0.061153	0.067*	0.5
H21E	0.813823	0.047438	0.018754	0.067*	0.5
C22	0.5932 (5)	0.0184 (4)	−0.0294 (3)	0.0485 (9)	
H22A	0.598140	0.106639	−0.101445	0.058*	
H22B	0.650354	−0.065663	−0.054982	0.058*	

### Atomic displacement parameters (Å^2^)

**Table:**

	*U*^11^	*U*^22^	*U*^33^	*U*^12^	*U*^13^	*U*^23^
Co1	0.02026 (19)	0.01482 (18)	0.01137 (18)	−0.00042 (13)	−0.00241 (14)	−0.00463 (13)
Br1	0.02518 (13)	0.02280 (13)	0.02382 (14)	0.00051 (9)	−0.01136 (10)	−0.00991 (10)
Br2	0.03810 (16)	0.01941 (13)	0.02232 (14)	−0.00291 (10)	0.00953 (10)	−0.01123 (10)
O1	0.0195 (7)	0.0143 (7)	0.0161 (7)	0.0058 (6)	−0.0053 (6)	−0.0070 (6)
N1	0.0196 (9)	0.0134 (8)	0.0159 (9)	0.0051 (7)	−0.0058 (7)	−0.0061 (7)
C1	0.0250 (11)	0.0177 (10)	0.0122 (10)	−0.0007 (8)	−0.0029 (8)	−0.0057 (8)
C2	0.0314 (13)	0.0187 (11)	0.0200 (11)	0.0045 (9)	−0.0055 (10)	−0.0064 (9)
C3	0.0334 (13)	0.0264 (12)	0.0235 (12)	0.0117 (10)	−0.0107 (10)	−0.0085 (10)
C4	0.0224 (11)	0.0291 (13)	0.0305 (13)	0.0070 (10)	−0.0087 (10)	−0.0136 (11)
C5	0.0201 (11)	0.0223 (11)	0.0235 (12)	0.0013 (9)	−0.0034 (9)	−0.0119 (9)
C6	0.0247 (11)	0.0173 (11)	0.0215 (11)	−0.0026 (9)	−0.0048 (9)	−0.0062 (9)
O11	0.0383 (10)	0.0193 (8)	0.0119 (7)	−0.0059 (7)	0.0046 (7)	−0.0063 (6)
N11	0.0373 (12)	0.0158 (9)	0.0139 (9)	−0.0049 (8)	0.0030 (8)	−0.0054 (7)
C11	0.0348 (13)	0.0173 (11)	0.0199 (12)	0.0049 (9)	−0.0010 (10)	−0.0056 (9)
C12	0.0414 (15)	0.0250 (13)	0.0210 (12)	0.0047 (11)	0.0042 (11)	−0.0067 (10)
C13	0.0589 (19)	0.0239 (12)	0.0161 (11)	−0.0058 (12)	0.0055 (12)	−0.0085 (10)
C14	0.063 (2)	0.0294 (14)	0.0200 (13)	−0.0174 (13)	−0.0001 (13)	−0.0112 (11)
C15	0.0461 (16)	0.0282 (13)	0.0240 (13)	−0.0153 (12)	−0.0010 (12)	−0.0119 (11)
C16	0.0295 (13)	0.0441 (16)	0.0257 (13)	0.0102 (12)	−0.0078 (11)	−0.0072 (12)
O21	0.053 (3)	0.044 (2)	0.044 (3)	0.006 (2)	−0.027 (2)	−0.022 (2)
C21	0.079 (3)	0.053 (2)	0.0378 (18)	0.035 (2)	−0.0255 (18)	−0.0177 (16)
C22	0.081 (3)	0.0339 (16)	0.0270 (15)	0.0242 (17)	−0.0127 (15)	−0.0121 (13)

### Geometric parameters (Å, º)

**Table:**

Co1—Br1	2.4312 (4)	C11—C12	1.391 (3)
Co1—Br2	2.5217 (4)	C11—C16	1.485 (4)
Co1—O1	1.9964 (16)	C12—H12	0.9500
Co1—O1^i^	2.1968 (15)	C12—C13	1.381 (4)
Co1—O11	1.9732 (17)	C13—H13	0.9500
O1—N1	1.357 (2)	C13—C14	1.368 (5)
N1—C1	1.357 (3)	C14—H14	0.9500
N1—C5	1.344 (3)	C14—C15	1.382 (4)
C1—C2	1.394 (3)	C15—H15	0.9500
C1—C6	1.480 (3)	C16—H16A	0.9800
C2—H2	0.9500	C16—H16B	0.9800
C2—C3	1.384 (4)	C16—H16C	0.9800
C3—H3	0.9500	O21—H21	0.8400
C3—C4	1.382 (4)	O21—C21	1.4524 (10)
C4—H4	0.9500	C21—H21A	0.9900
C4—C5	1.380 (3)	C21—H21B	0.9900
C5—H5	0.9500	C21—H21C	0.9800
C6—H6A	0.9800	C21—H21D	0.9800
C6—H6B	0.9800	C21—H21E	0.9800
C6—H6C	0.9800	C21—C22	1.539 (5)
O11—N11	1.347 (2)	C22—C22^ii^	1.528 (8)
N11—C11	1.346 (4)	C22—H22A	0.9900
N11—C15	1.351 (4)	C22—H22B	0.9900
			
Br1—Co1—Br2	101.355 (16)	N11—C11—C12	117.5 (3)
O1—Co1—Br1	116.56 (5)	N11—C11—C16	118.2 (2)
O1^i^—Co1—Br1	95.39 (4)	C12—C11—C16	124.3 (3)
O1—Co1—Br2	94.52 (4)	C11—C12—H12	119.5
O1^i^—Co1—Br2	161.54 (4)	C13—C12—C11	121.1 (3)
O1—Co1—O1^i^	70.82 (7)	C13—C12—H12	119.5
O11—Co1—Br1	112.08 (6)	C12—C13—H13	120.4
O11—Co1—Br2	97.54 (5)	C14—C13—C12	119.3 (2)
O11—Co1—O1^i^	83.12 (6)	C14—C13—H13	120.4
O11—Co1—O1	126.08 (7)	C13—C14—H14	120.2
Co1—O1—Co1^i^	109.18 (7)	C13—C14—C15	119.5 (3)
N1—O1—Co1	125.78 (12)	C15—C14—H14	120.2
N1—O1—Co1^i^	124.80 (12)	N11—C15—C14	119.7 (3)
O1—N1—C1	118.60 (18)	N11—C15—H15	120.1
C5—N1—O1	118.18 (19)	C14—C15—H15	120.1
C5—N1—C1	123.2 (2)	C11—C16—H16A	109.5
N1—C1—C2	117.5 (2)	C11—C16—H16B	109.5
N1—C1—C6	118.4 (2)	C11—C16—H16C	109.5
C2—C1—C6	124.0 (2)	H16A—C16—H16B	109.5
C1—C2—H2	119.8	H16A—C16—H16C	109.5
C3—C2—C1	120.5 (2)	H16B—C16—H16C	109.5
C3—C2—H2	119.8	C21—O21—H21	109.5
C2—C3—H3	120.2	O21—C21—H21A	109.2
C4—C3—C2	119.7 (2)	O21—C21—H21B	109.2
C4—C3—H3	120.2	O21—C21—C22	112.1 (3)
C3—C4—H4	120.4	H21A—C21—H21B	107.9
C5—C4—C3	119.2 (2)	H21C—C21—H21D	109.5
C5—C4—H4	120.4	H21C—C21—H21E	109.5
N1—C5—C4	119.9 (2)	H21D—C21—H21E	109.5
N1—C5—H5	120.1	C22—C21—H21A	109.2
C4—C5—H5	120.1	C22—C21—H21B	109.2
C1—C6—H6A	109.5	C22—C21—H21C	109.5
C1—C6—H6B	109.5	C22—C21—H21D	109.5
C1—C6—H6C	109.5	C22—C21—H21E	109.5
H6A—C6—H6B	109.5	C21—C22—H22A	108.9
H6A—C6—H6C	109.5	C21—C22—H22B	108.9
H6B—C6—H6C	109.5	C22^ii^—C22—C21	113.3 (3)
N11—O11—Co1	120.09 (13)	C22^ii^—C22—H22A	108.9
O11—N11—C15	118.3 (2)	C22^ii^—C22—H22B	108.9
C11—N11—O11	118.8 (2)	H22A—C22—H22B	107.7
C11—N11—C15	122.9 (2)		
			
Co1^i^—O1—N1—C1	102.20 (19)	C5—N1—C1—C6	−175.8 (2)
Co1—O1—N1—C1	−84.0 (2)	C6—C1—C2—C3	176.9 (2)
Co1^i^—O1—N1—C5	−79.2 (2)	O11—N11—C11—C12	−177.4 (2)
Co1—O1—N1—C5	94.6 (2)	O11—N11—C11—C16	3.0 (3)
Co1—O11—N11—C11	−111.6 (2)	O11—N11—C15—C14	177.7 (2)
Co1—O11—N11—C15	69.9 (3)	N11—C11—C12—C13	−0.9 (4)
O1—N1—C1—C2	−179.10 (19)	C11—N11—C15—C14	−0.7 (4)
O1—N1—C1—C6	2.8 (3)	C11—C12—C13—C14	0.5 (4)
O1—N1—C5—C4	179.4 (2)	C12—C13—C14—C15	−0.1 (4)
N1—C1—C2—C3	−1.1 (3)	C13—C14—C15—N11	0.2 (4)
C1—N1—C5—C4	−2.1 (4)	C15—N11—C11—C12	1.1 (4)
C1—C2—C3—C4	−0.3 (4)	C15—N11—C11—C16	−178.6 (2)
C2—C3—C4—C5	0.6 (4)	C16—C11—C12—C13	178.7 (3)
C3—C4—C5—N1	0.5 (4)	O21—C21—C22—C22^ii^	−79.5 (5)
C5—N1—C1—C2	2.3 (3)		

Symmetry codes: (i) −*x*+1, −*y*+1, −*z*+1; (ii) −*x*+1, −*y*, −*z*.

### Hydrogen-bond geometry (Å, º)

**Table:**

*D*—H···*A*	*D*—H	H···*A*	*D*···*A*	*D*—H···*A*
C5—H5···Br1^iii^	0.95	3.12	3.704 (2)	121
C5—H5···Br1^i^	0.95	2.98	3.531 (2)	118
C6—H6*A*···O11^i^	0.98	2.45	3.259 (3)	140
C6—H6*B*···Br2^iv^	0.98	2.88	3.852 (2)	172
C6—H6*C*···Br1	0.98	3.02	3.845 (2)	142
C12—H12···Br2^v^	0.95	2.99	3.676 (3)	130
C12—H12···O21^v^	0.95	2.55	3.441 (6)	156
C14—H14···Br1^vi^	0.95	3.11	3.747 (3)	126
C16—H16*C*···Br1^iii^	0.98	3.13	3.725 (3)	121
O21—H21···Br2	0.84	2.43	3.244 (4)	162

Symmetry codes: (i) −*x*+1, −*y*+1, −*z*+1; (iii) *x*+1, *y*, *z*; (iv) −*x*+1, −*y*, −*z*+1; (v) −*x*+2, −*y*+1, −*z*; (vi) −*x*+1, −*y*+1, −*z*.
