# Supplementary figures and images for: Synthesis, crystal structure and reactivity of bis­(μ-2-methyl­pyridine N-oxide-κ2 O:O)bis­[di­bromido­(2-methyl­pyridine N-oxide-κO)cobalt(II)] butanol monosolvate
Source: Acta Crystallogr E Crystallogr Commun. 2023 Oct 3;79(Pt 11):972–6. doi: 10.1107/S2056989023008228 (PMC10626943; doi:10.1107/S2056989023008228)

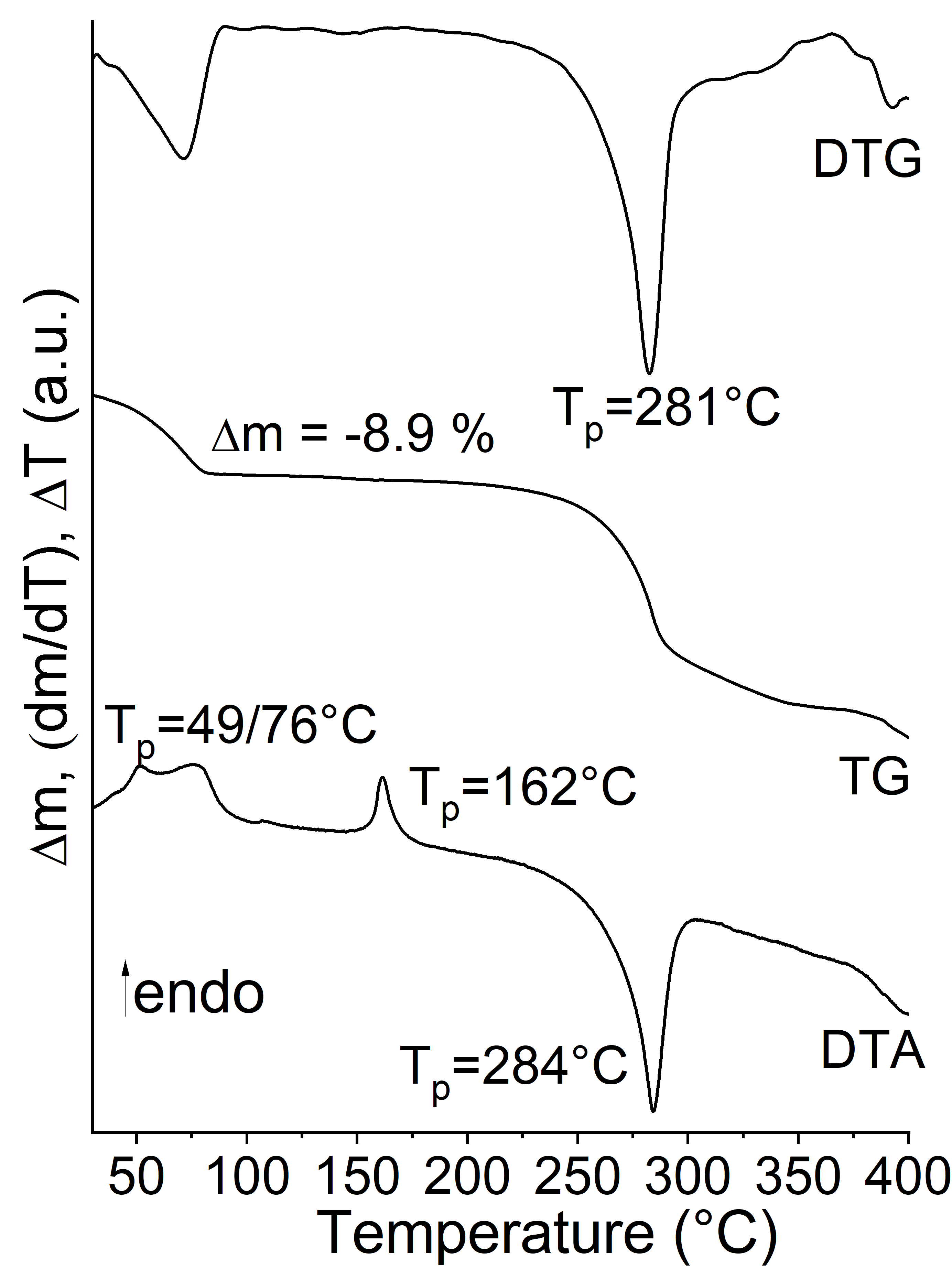

Supplement: Supplementary file 3 [file e-79-00972-sup3.png]

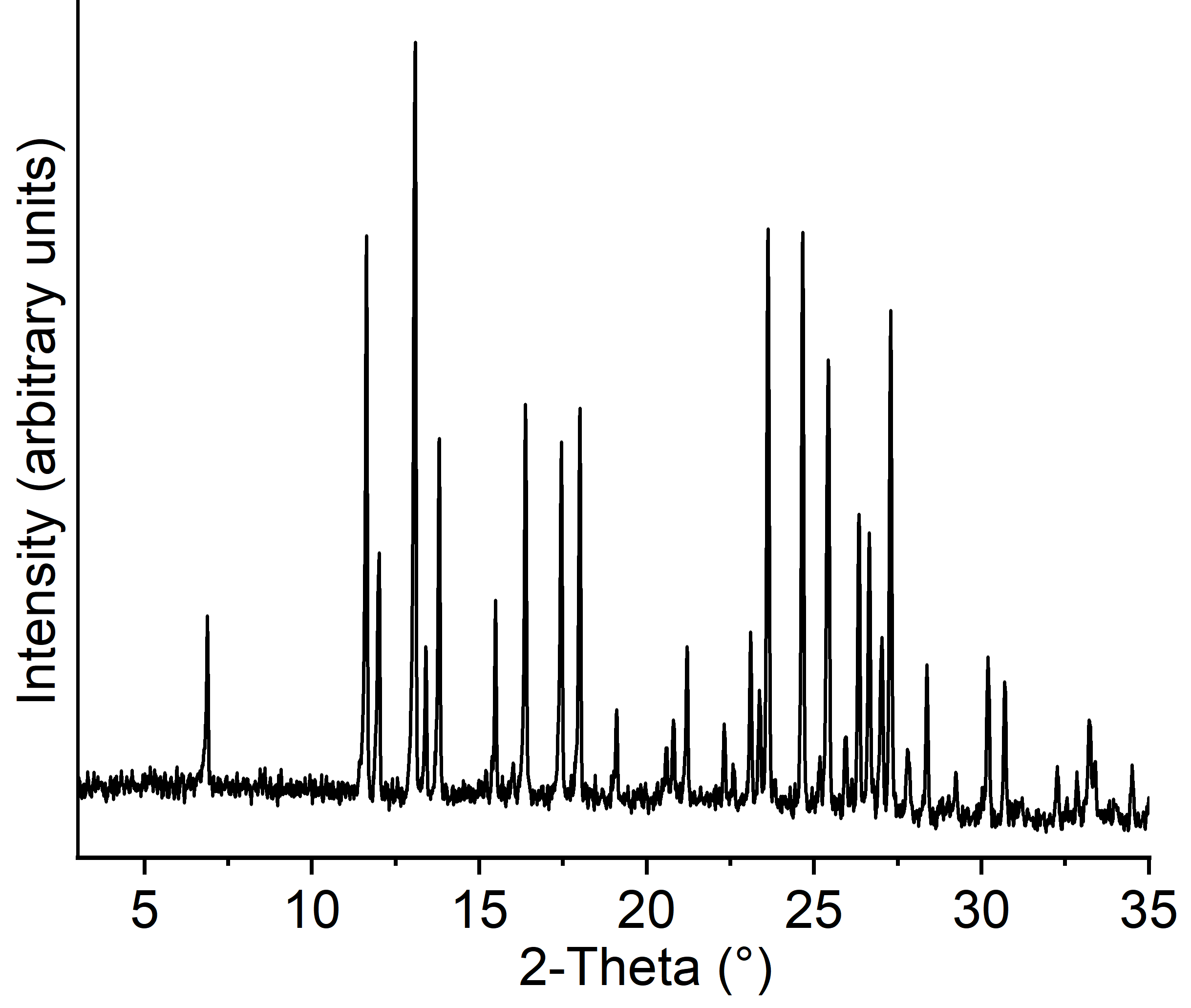

Supplement: Supplementary file 4 [file e-79-00972-sup4.png]

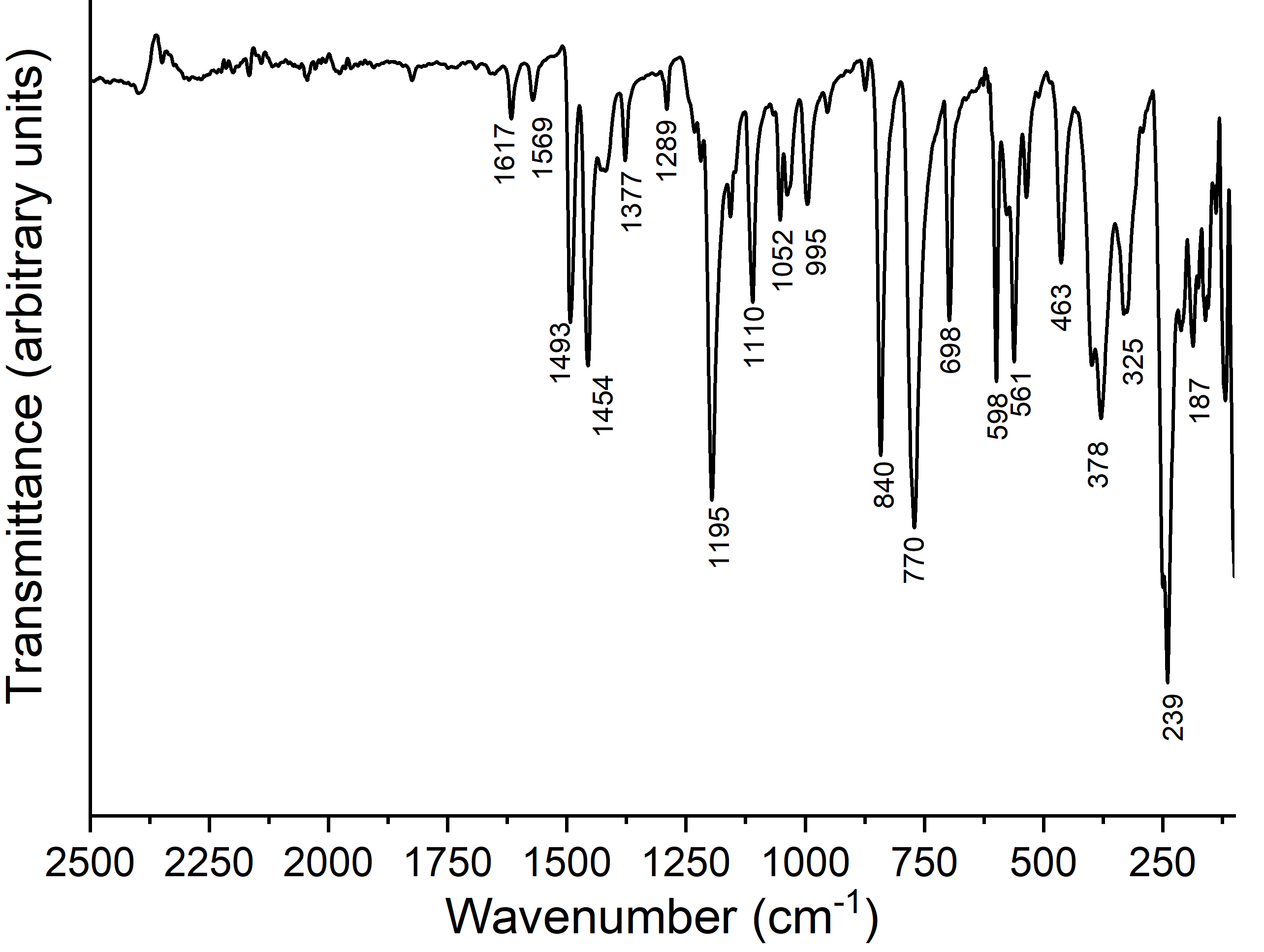

Supplement: Supplementary file 5 [file e-79-00972-sup5.png]

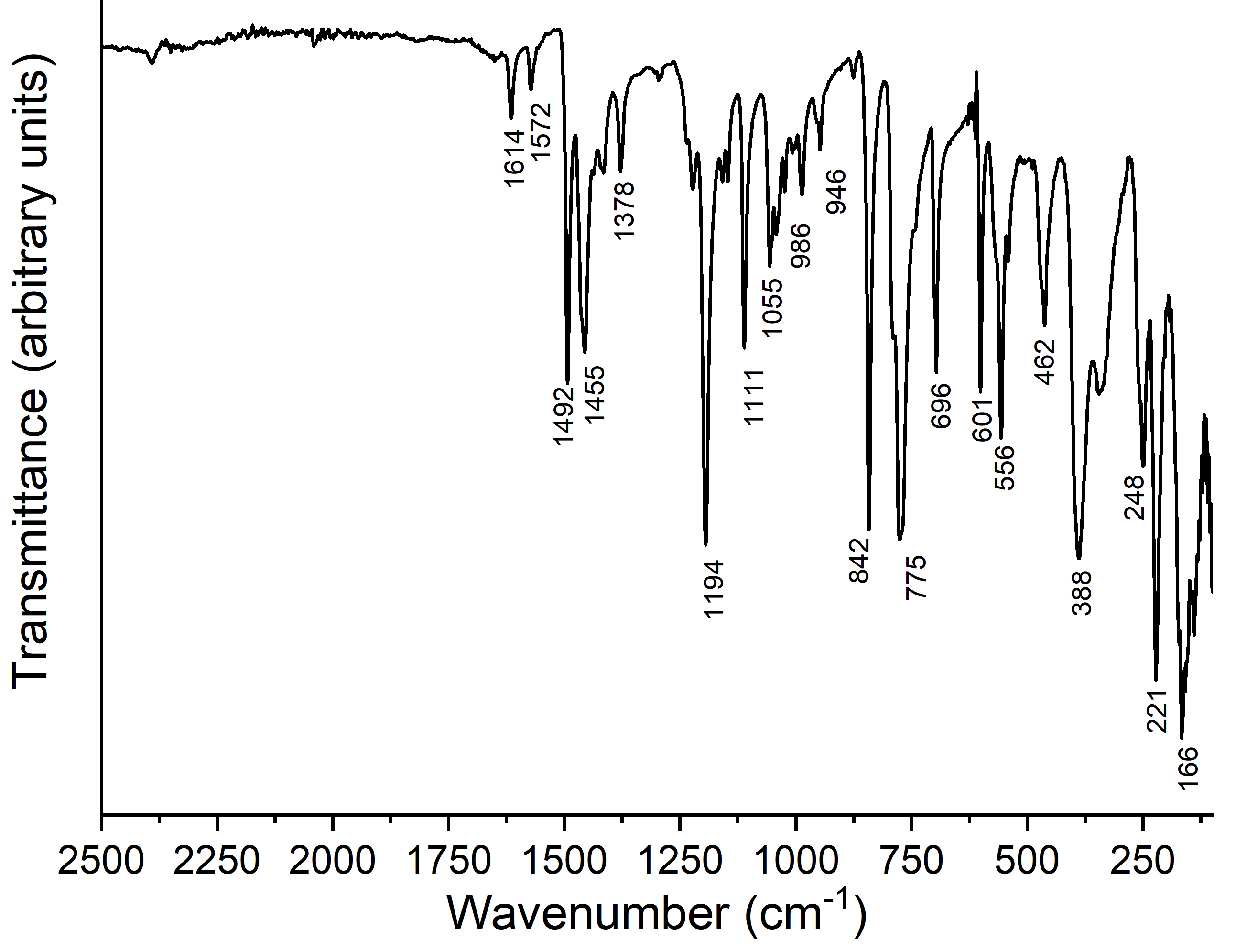

Supplement: Supplementary file 6 [file e-79-00972-sup6.png]

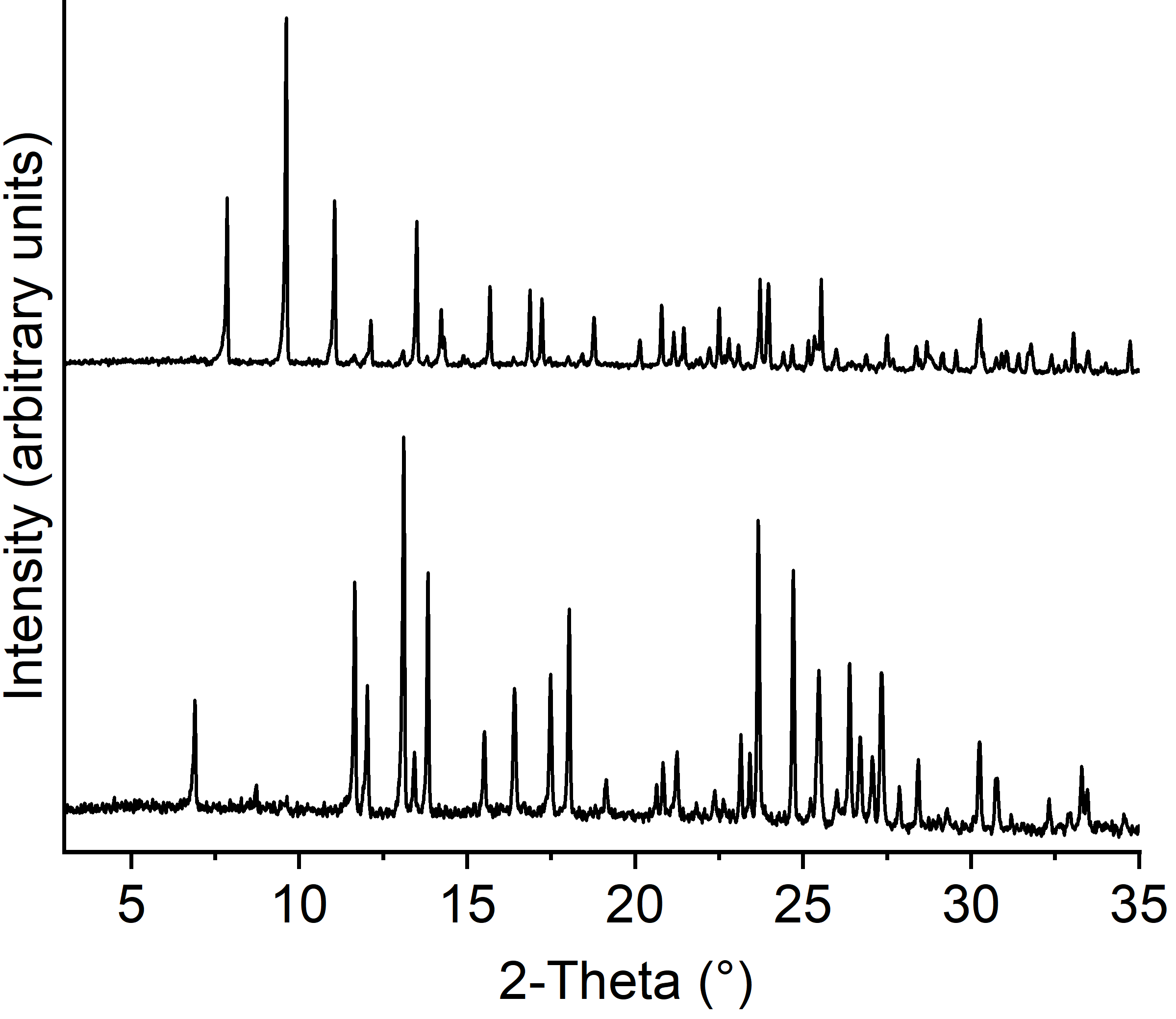

Supplement: Supplementary file 7 [file e-79-00972-sup7.png]
